# Vocal Tract Articulation in Zebra Finches

**DOI:** 10.1371/journal.pone.0011923

**Published:** 2010-07-30

**Authors:** Verena R. Ohms, Peter Ch. Snelderwaard, Carel ten Cate, Gabriël J. L. Beckers

**Affiliations:** 1 Behavioural Biology, Institute of Biology Leiden, Leiden University, Leiden, The Netherlands; 2 Leiden Institute for Brain and Cognition, Leiden University, Leiden, The Netherlands; 3 Behavioural Neurobiology, Max Planck Institute for Ornithology, Seewiesen, Germany; University of Sussex, United Kingdom

## Abstract

**Background:**

Birdsong and human vocal communication are both complex behaviours which show striking similarities mainly thought to be present in the area of development and learning. Recent studies, however, suggest that there are also parallels in vocal production mechanisms. While it has been long thought that vocal tract filtering, as it occurs in human speech, only plays a minor role in birdsong there is an increasing number of studies indicating the presence of sound filtering mechanisms in bird vocalizations as well.

**Methodology/Principal Findings:**

Correlating high-speed X-ray cinematographic imaging of singing zebra finches (*Taeniopygia guttata*) to song structures we identified beak gape and the expansion of the oropharyngeal-esophageal cavity (OEC) as potential articulators. We subsequently manipulated both structures in an experiment in which we played sound through the vocal tract of dead birds. Comparing acoustic input with acoustic output showed that OEC expansion causes an energy shift towards lower frequencies and an amplitude increase whereas a wide beak gape emphasizes frequencies around 5 kilohertz and above.

**Conclusion:**

These findings confirm that birds can modulate their song by using vocal tract filtering and demonstrate how OEC and beak gape contribute to this modulation.

## Introduction

Birdsong is a complex vocal behaviour often considered to show striking developmental and structural similarities with human speech [1]. However, these similarities are mainly thought to be present in the area of development and learning whereas vocal production mechanisms have long been considered to be fundamentally different.

In humans, voiced speech is produced by vibrations of the vocal folds which are subsequently filtered in order to produce different speech sounds that form an important part of our phonetic repertoire [2]. This filtering process takes place in the upper vocal tract by altering the dimensions of various resonance cavities within the vocal tract, like pharyngeal, oral and nasal cavity. This is achieved by moving articulators such as tongue, lips and lower jaw [3].

In contrast to this source-filter theory of human speech [4] it has been long thought that frequency and amplitude modulations of bird vocalizations are mainly produced by the avian sound source, the syrinx, and that vocal tract filtering as in human speech production plays a minor role in generating vocal complexity in birdsong [5]. However, recent studies suggest that this view needs to be reconsidered as there is a growing body of evidence indicating the significance of vocal tract filtering in bird vocal communication as well.

Some of the first evidence derives from experiments showing that both songbirds and non-songbirds singing in heliox exhibit deviating vocal characteristics [6], [7]. Harmonic overtones of supposedly pure tones become apparent as well as a shifted emphasis towards higher frequencies in broad-band sounds. These observations lead to the hypothesis that the bird's vocal tract can act as an acoustic filter and be actively modulated [6]. Motivated by these findings subsequent studies on potential vocal tract articulators showed that beak movements and gape width are correlated with frequency patterns in white-throated sparrows (*Zonotrichia albicollis*), swamp sparrows (*Melospiza georgiana*) [8], Darwin's finches [9] and zebra finches (*Taeniopygia guttata*) [10]. Furthermore, in zebra finches correlations between beak gape and amplitude have been found [10], [11]. Experimentally manipulating beak movements and gape widths also affects frequency patterns in white-throated sparrows, swamp sparrows, canaries (*Serinus canaria*) [12] and zebra finches [11].

Other studies indicate that expanding the oropharyngeal-esophageal cavity (OEC) plays a role in vocal tract filtering as well by tuning it to the fundamental frequency of the vocalizations in doves (*Streptopelia risoria*) [13], northern cardinals (*Cardinalis cardinalis*) [14] and white-throated sparrows [15]. Tongue movements in monk parakeets (*Myiopsitta monachus*) also seem to have a filtering effect on the sound produced [16].

Although all of the mentioned studies suggest that possible articulators such as beak and the expandable esophagus are likely to modulate birdsong, these data are predominantly correlational. As such, they are insufficient to precisely assess the role of different articulators in vocal production since their effects usually cannot be separated from each other or from other factors such as variation at the sound source. In the current study we combined correlational and experimental data on vocal production in zebra finches. First we used high-speed X-ray cinematographic imaging to quantify patterns of both beak movements and OEC expansion during singing (Video S1) and matched these patterns to distinct note types. Subsequently we conducted an experiment in which we replaced the syrinx by a mini-loudspeaker and played frequency sweeps under varying articulator configurations through the vocal tract (similar to [16]). We manipulated beak gape and OEC expansion and compared acoustic input with acoustic output in order to evaluate the significance of these possible articulators.

## Materials and Methods

### (a) Ethics Statement

All animals came from the Leiden University breeding colony and were housed in groups of at least two birds prior to the experiments. All animal procedures were approved by the animal experimentation committee of Leiden University (DEC numbers 08116 and 07190).

### (b) Subjects

We used five male and two female zebra finches for the X-ray cinematographic imaging and five male zebra finches for the experiment in which we replaced the syrinx by a mini-loudspeaker. The female birds only served as stimulus birds in the X-ray setting to stimulate the males to sing. During X-ray recordings male birds were individually transferred into a small cage (30 cm wide ×20 cm high ×10 cm deep) built from wood with plexi glass on both long sides. The small size of the cage allowed to optically focus on the birds, but still allowed the typical dancing movements during singing [10].

### (c) Cineradiography

A Philips Optimus M 200 X-ray apparatus was combined with a Kodak Motion Corder Analyzer SR- 500 s that records at 500 field s^−1^, shutter speed 1/500 s by replacing the original camera of the X-ray apparatus by the Kodak system. The images which had a resolution of 512×240 pixels were loaded into the camera's onboard memory. The maximum recording time of the Kodak Motion Corder which was triggered manually is 8.7 s at 500 fields s^−1^, making it necessary to save the video sequences immediately on digital video for permanent storage [17]. For that we used a Sony Mini Digital Video cassette recorder Model No. GV-D900E and later on an LG DVD Player (DVD Player ± RW Recorder) Model No. DR6621 on which simultaneously sound was recorded too using a pre-amplifier (Marantz PMD661) and a directional microphone (Sennheiser ME 67/K6) aimed at the bird from 0.5 m distance. As these devices have a frame rate of only 25 frames s^−1^ we played back the video sequences from the Kodak system with 25 frames s^−1^ to prevent data loss while re-recording. We continuously applied an X-ray dose of 56 kV, 60 mA. The videos were captured either from the Mini DV tapes or from the DVDs using Adobe Premiere Pro software version 7.0 for Windows. Due to a distinct tone produced by the X-ray apparatus only while the shutter was open it was possible to align sound and video using the frame-matching features of Adobe Premiere with an accuracy of 2 milliseconds. In order to accurately follow and quantify the movements of certain articulators we also glued several lead markers (ca. 0.5 mm^3^) on head and beak of the birds using tissue adhesive (Superglue 90–120 CPS, World Precision Instruments, Inc., Sarasota, Florida, USA). In two birds we implanted additional lead markers into the tongue and larynx. These procedures were conducted under anesthesia using isoflurane (1.8%, O_2_ 0.3 l/min, N_2_O 0.4 l/min).

To quantify beak gape and OEC expansion we measured the distance between the tips of mandible and maxilla and the distance between the most ventral point of the OEC and the midpoint of the neck of the bird for several note types per song and always at the temporal midpoint of those notes as identified on song spectrograms. These measurements were taken from still images using AviDigitiser (© Peter Ch. Snelderwaard) which provides the coordinates of manually selected points within each video frame and from which distances can be calculated. Only X-ray images in which the birds kept their head in a perfect lateral position towards the camera were used, so that in the end we obtained measurements from at least 8 songs for every measured note type. Although we recorded with a high frame rate, the stereotyped dancing movements of zebra finches allowed sampling of only a few notes per song per bird, namely those in which the bird's head was perfectly lateral to the camera. This method is well suited for comparing beak gape and OEC expansion for various elements within a song, as well as for qualitative comparison between birds, but not for quantitative comparisons between birds. Furthermore, due to the small size of zebra finches it was not possible to identify structures such as single vertebrae, but the overall shape of OEC and neck was used to obtain measurements.

Afterwards we carried out a direct discriminant function analysis using beak gape and OEC expansion as predictors for determining which note types were produced. Since zebra finches produce several different and complex note types we did not relate our articulation measurements to signal analytic features such as fundamental or peak frequency as has been done in other studies [14]–[15]. Little is known about how different zebra finch note types are produced physiologically, but it seems likely that they correspond to different syringeal production modes. Correlating parameters such as fundamental frequency with vocal tract articulation should therefore not be based on an analysis that mixes replicate notes of different types, but rather one that distinguishes within- and between note type variation. For such an analysis, however, more data would be necessary. At the same time articulatory states of beak gape and OEC expansion might be different enough between various note types to allow predicting which note types relate to different articulator configurations using a discriminant function analysis, although note types exhibiting similar articulatory modes are less likely to be classified correctly.

Prior to the X-ray cinematography we recorded the songs of each bird in a sound-attenuating chamber (ca. 1.80 m ×1.20 m ×2.00 m) that was lined with acoustic foam (Gamma geluidsisolatie platen product number 102247, Intergamma B.V. Leusden, The Netherlands) to reduce sonic reflections from the walls. From these recordings we later took amplitude measurements using the software PRAAT (version 4.6.09, freely available at www.praat.org) [18] of those note types for which we also measured beak gape and OEC expansion on the X-ray videos. We took care to always take measurements from the temporal midpoint of each note as identified on sound spectrograms in both X-ray videos and song recordings.

However, since X-ray cinematography does not allow evaluating the effects of beak gape and OEC expansion separately from the sound source the second experiment was conducted to assess a causal relationship and to examine the role of each of these structures in vocal tract filtering directly.

### (d) Speaker experiment

One observation made on the X-ray videos is that OEC expansion is caused by a posterior-ventral movement of the hyoid skeleton. Therefore we posterior-ventrally displaced the hyoid skeleton in 0.5 mm steps to gradually increase OEC expansion and evaluate its filtering characteristics while playing frequency sweeps through the vocal tract of freshly sacrificed zebra finches. We did so for three different beak gapes.

The birds used for this experiment were euthanized with an overdose of Nembutal (300 mg/kg body weight) in the pectoral muscle. Afterwards a small incision was made posterior from the lower jaw to expose the urohyal bone [19] of the tongue apparatus. A cord was knotted around this bone which was later attached to a micromanipulator that could be moved in 0.5 millimeter steps. Subsequently, the syrinx and a part of the trachea were made accessible by dissecting the birds ventrally between the clavicles following the sternum. The trachea was intersected just above the splitting into the two primary bronchi and a short silastic tube which was fitted over the port of a small speaker (Knowles WBHC NB-68438C, Itasca, Illinois, USA) was inserted into the trachea so that the speaker was placed in the same position where otherwise the syrinx would have been [16]. The dissected tissue was then agglutinated with tissue adhesive (Superglue 90–120 CPS, World Precision Instruments, Inc., Sarasota, Florida, USA) and the head of the bird was fixed in a stereotaxic device in such a way that the bill was positioned vertically. A thin metal wire (0.7 mm diameter) was stuck between the tips of mandible and maxilla and fixed with tissue adhesive to keep the beak gape constant. During the experiment acoustic measurements with three different beak gapes were taken whereas in the first series the beak was kept open at ca. 4.0 millimeters which represented a wide opening as observed on the X-ray videos only during some notes. In the second series the beak was kept open at 1.0 millimeter, a range frequently observed during natural zebra finch song. In the third series the beak was closed completely. Within each series the position of the hyoid skeleton was changed stepwise by displacing the urohyal bone ventrally in 0.5 millimeter steps in order to model the expansion of the OEC as observed on the X-ray videos. The maximal ventral movement of the urohyal bone varied between birds and series with a minimal displacement of 4.0 millimeters and a maximal displacement of 6.5 millimeters. The acoustic measurements took place in the sound-attenuating chamber described above. For every position of the tongue apparatus within all three series a linear frequency sweep (0.3 to 10 kHz in 1 second) constructed with PRAAT was played through the vocal tract of the birds using a sound card (CDX-01 CardDeluxe, Digital Audio Labs, 1266 Park Road Chanhassen, MN 55317). The sound emitted from the beak was then recorded with a Sennheiser MKH50 microphone vertically directed at the beak from 3 cm distance and immediately recorded in PRAAT with the same sound card (44.1 kilosamples/s, 16 bit resolution). After the experiment we checked for every bird whether the speaker was still attached to the trachea, which was the case for all five birds. To ensure that differences between spectra of recorded sweeps were caused by differences in articulation and not by position-dependent filtering due to remaining room resonances, we took care that the exact position of both the microphone and the bird preparation did not change between recordings. We also measured speaker output at approximately the same position where the beak was during recordings in order to correct for frequency response deviations of the speaker system by subtracting the dB values of the speaker output from the measured spectrum. Although remaining resonances might still affect the data slightly this impact can be considered rather small and does not change the general results. The data were analyzed by calculating the long-time average spectrums (Ltas function in PRAAT; 100 Hz bin width) of the recorded sound sweeps, and comparing them between different articulatory states. The latter was done using custom-written scripts in the scientific computing environment SciPy version 0.7 [20].

## Results

### (a) Cineradiography

**Figure 1:**
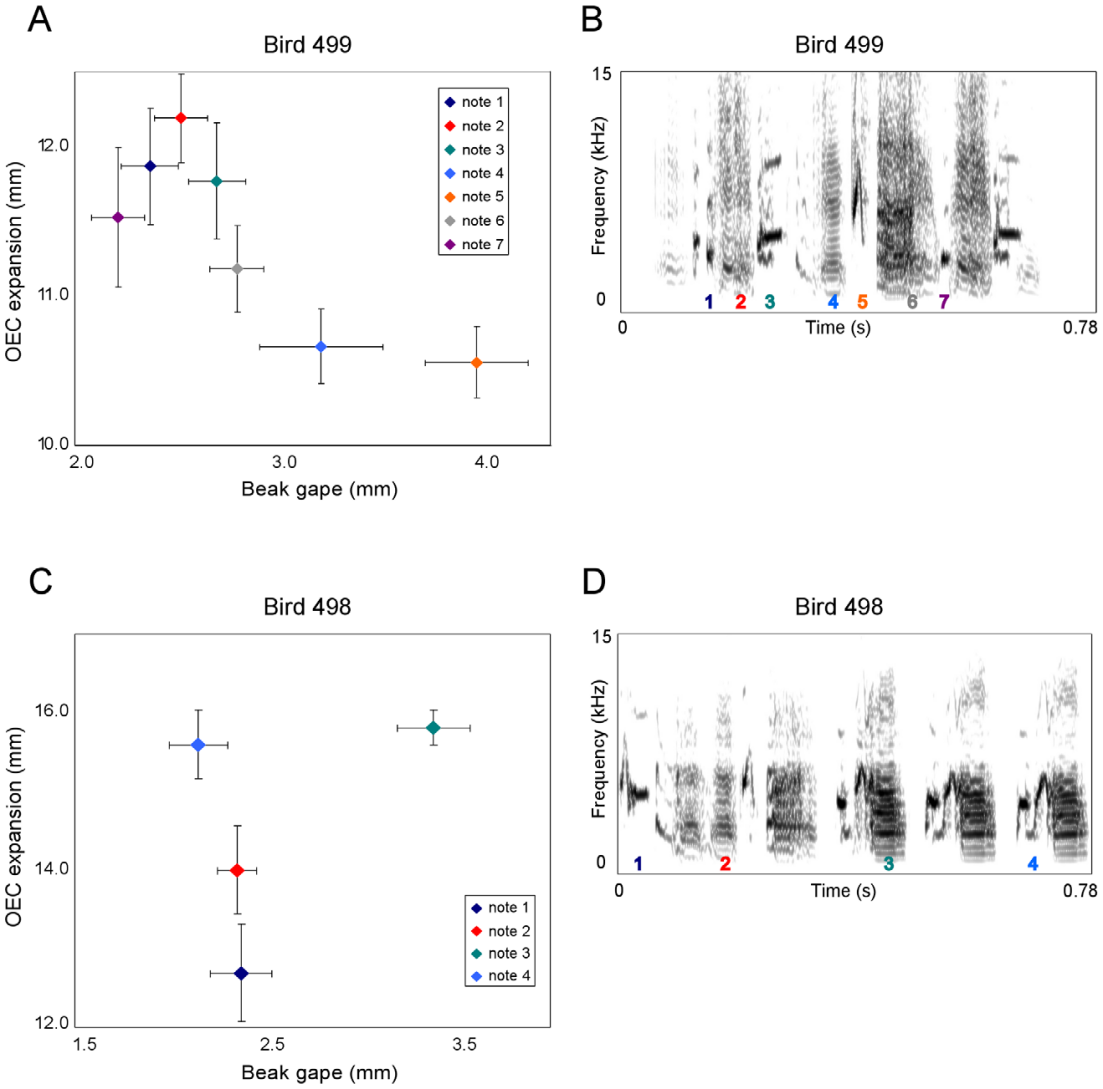
Scatter plots of measured note types and song spectrograms for birds 499 and 498. This figure illustrates the results of the measurements taken from the X-ray videos of birds 499 and 498. In panels (a) and (c) average OEC expansion (in millimeters) is plotted against average beak gape (in millimeters) including standard error for several distinct note types measured from at least 8 songs per note. Panels (b) and (d) show the associated spectrograms. The numbers below the notes in the spectrograms correspond to the plotted notes in panels (a) and (c). mm, millimeters; kHz, kilohertz; s, seconds; OEC, oropharyngeal-esophageal cavity.

**Figure 2:**
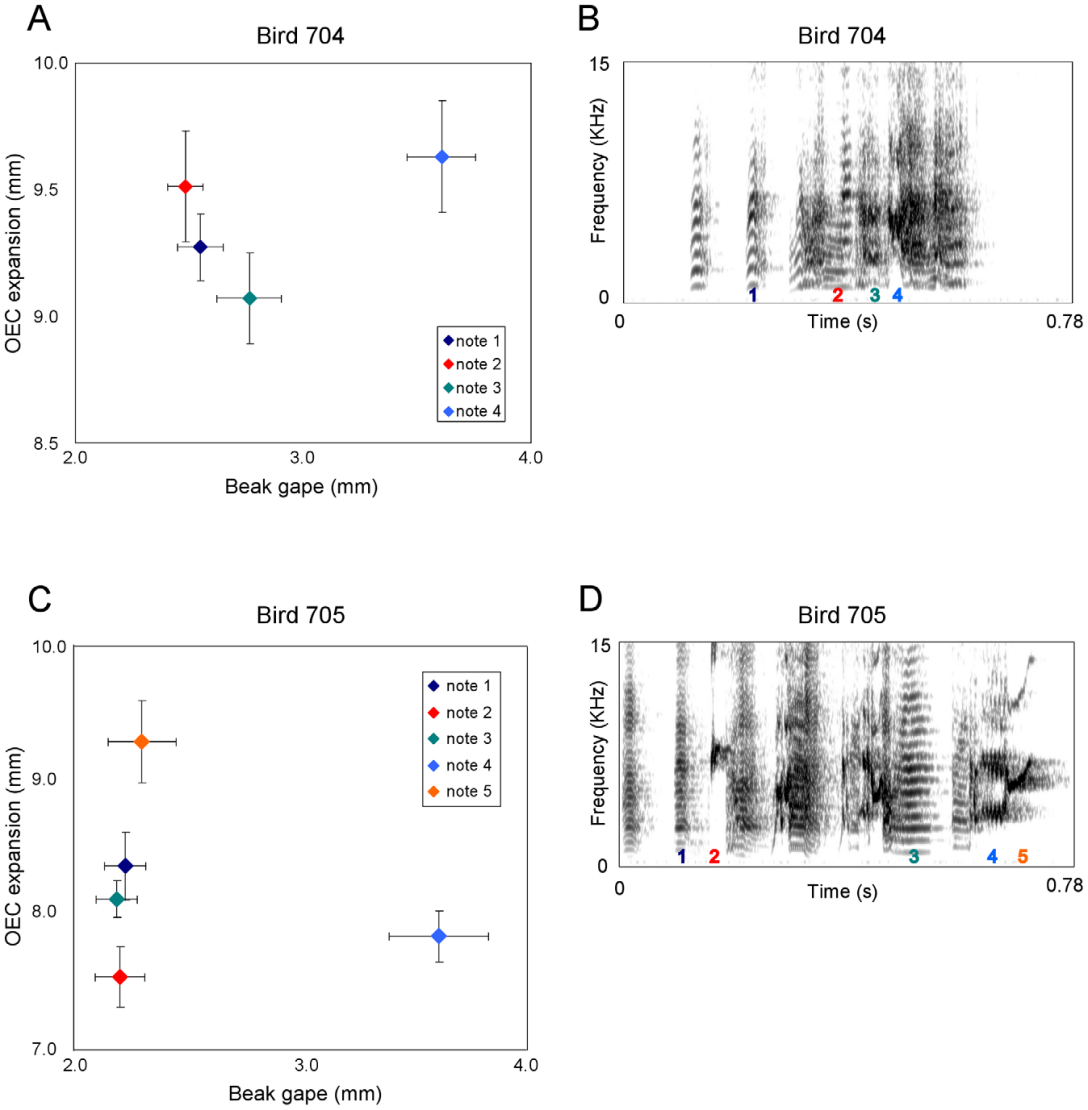
Scatter plots of measured note types and song spectrograms for birds 704 and 705. This figure is equivalent to figure 1 and shows the results of the measurements taken from the X-ray videos of the remaining two birds. Again panels (a) and (c) give scatter plots of average beak gape versus average OEC expansion including standard error with the associated spectrograms in (b) and (d). mm, millimeters; kHz, kilohertz; s, seconds; OEC, oropharyngeal-esophageal cavity.

We obtained sufficient video data from four male birds and measured beak gape and OEC expansion of different note types within each song per individual. Figures 1 and 2 suggest that different note types are characterized by different combinations of beak gape and OEC expansion.

For every bird separately a direct discriminant function analysis was carried out (Tables 1–3) with beak gape and OEC expansion as predictors for distinct note types. Two discriminant functions were calculated both of which are significant in birds 498 and 705. In the other two birds, 499 and 704, only the first, but not the second, discriminant functions are significant (Table 1).

**Table 1:** Statistical significance of discriminant functions.

Bird	Test of function(s)	Wilks' Lambda	Chi-square	df	p
498	1 through 2	0.221	42.224	6	**0.000**
	2	0.517	18.478	2	**0.000**
499	1 through 2	0.542	68.993	12	**0.000**
	2	0.959	4.753	5	0.447
704	1 through 2	0.573	50.736	6	**0.000**
	2	0.962	3.480	2	0.176
705	1 through 2	0.283	91.443	8	**0.000**
	2	0.722	23.611	3	**0.000**

This table gives Wilks' lambda for the two discriminant functions, using beak gape and OEC expansion as parameters, calculated for every bird separately and the chi-square values into which Wilks' lambda can be transformed as well as the corresponding p values. Significant p values are printed bold. df, degrees of freedom.

**Table 2 pone-0011923-t002:** Discriminant function coefficients and within-group correlations.

		Coefficients		Correlation	
Bird	Variable	Function 1	Function 2	Function 1	Function 2
498	Beak gape	0.994	−0.416	1.000	−0.014
	OEC expansion	0.16	1.078	0.386	0.922
499	Beak gape	0.910	0.430	0.850	0.527
	OEC expansion	−0.530	−0.855	−0.427	0.904
704	Beak gape	1.012	−0.088	0.996	0.092
	OEC expansion	−0.093	1.012	0.087	0.996
705	Beak gape	0.974	0.305	0.876	0.482
	OEC expansion	−0.492	0.894	−0.299	0.954

This table lists the standardized canonical discriminant function coefficients and the pooled within-groups correlations between discriminating variables (beak gape and OEC expansion) and both discriminant functions for every individual bird. In all four birds beak gape is the larger standardized coefficient in the first discriminant function and also has the stronger correlation, whereas in the second discriminant function OEC expansion is the larger standardized coefficient and also shows the stronger correlation. OEC, oropharyngeal-esophageal cavity.

**Table 3:** Classification results.

Bird 498						
	Predicted group membership in % (counts)					
Note type	1	2	3	4	N	Chance (%)
**1**	62.5 (5)	25.0 (2)	12.5 (1)	0.0 (0)	8	25
**2**	37.5 (3)	25.0 (2)	0.0 (0)	37.5 (3)	8	25
**3**	0.0 (0)	0.0 (0)	100.0 (8)	0.0 (0)	8	25
**4**	0.0 (0)	12.5 (1)	0.0 (0)	87.5 (7)	8	25

Bird 704						
	Predicted group membership in % (counts)					
Note type	1	2	3	4	N	Chance (%)
**1**	40.0 (10)	32.0 (8)	24.0 (6)	4.0 (1)	25	26.3
**2**	29.2 (7)	58.3 (14)	8.3 (2)	4.2 (1)	24	25.3
**3**	23.8 (5)	14.3 (3)	28.6 (6)	33.3 (7)	21	22.1
**4**	0.0 (0)	12.0 (3)	12.0 (3)	76.0 (19)	25	26.3

Bird 705							
	Predicted group membership in % (counts)						
Note type	1	2	3	4	5	N	Chance (%)
**1**	0.0 (0)	23.1 (3)	38.5 (5)	0.0 (0)	38.5 (5)	13	16.9
**2**	0.0 (0)	60.0 (9)	26.7 (4)	6.7 (1)	6.7 (1)	15	19.5
**3**	0.0 (0)	17.6 (3)	64.7 (11)	0.0 (0)	17.6 (3)	17	22.1
**4**	0.0 (0)	12.5 (2)	6.3 (1)	81.3 (13)	0.0 (0)	16	20.8
**5**	0.0 (0)	12.5 (2)	18.8 (3)	6.3 (1)	62.5 (10)	16	20.8

Bird 499									
	Predicted group membership in % (counts)								
Note type	1	2	3	4	5	6	7	N	Chance (%)
**1**	17.6 (3)	41.2 (7)	0.00 (0)	17.6 (3)	0.0 (0)	0.0 (0)	23.5 (4)	17	14.4
**2**	5.9 (1)	41.2 (7)	11.8 (2)	5.9 (1)	0.0 (0)	11,8 (2)	23.5 (4)	17	14.4
**3**	5.9 (1)	29.4 (5)	17.6 (3)	11.8 (2)	0.0 (0)	17.6 (3)	17.6 (3)	17	14.4
**4**	0.0 (0)	6.3 (1)	6.3 (1)	6.3 (1)	31.3 (5)	31.3 (5)	18.8 (3)	16	13.6
**5**	0.0 (0)	0.0 (0)	5.9 (1)	5.9 (1)	70.6 (12)	11.8 (2)	5.9 (1)	17	14.4
**6**	5.9 (1)	17.6 (3)	11.8 (2)	11.8 (2)	11.8 (2)	29.4 (5)	11.8 (2)	17	14.4
**7**	5.9 (1)	29.4 (5)	0.0 (0)	17.6 (3)	0.0 (0)	5.9 (1)	41.2 (7)	17	14.4

In this table the percentages as well as the actual numbers of cases in which a note type has been correctly classified as belonging to it's own group or misclassified as belonging to another note type are given for each individual bird. In the last column the percentage for a certain note type being correctly identified by chance is listed.

In all four birds beak gape is weighted heavier in the first discriminant function whereas OEC expansion is weighted more in the second, as shown by the standardized coefficients and the correlation between each variable and any discriminant function (Table 2).

The classification results (Table 3) show that the percentage of cases in which note types were correctly classified as belonging to their own group is generally well above the percentage expected by chance although some note types were not correctly assigned. Especially in bird 499 (Fig. 1, Table 3) in which seven different note types were measured the classification for three of these note types remained around chance level, whereas in the other three birds always one note type appeared to be difficult to assign to the right group. In bird 705 note 1 was never properly allocated (Fig. 2, Table 3) which can be explained by the large overlap between this note and notes 3 and 5. However, the average value of note 1 is closer to the average value of note 3 compared to 5 while at the same time both notes represent harmonic stacks with a comparable sound shape. In bird 499 notes 1 and 3 show a similar structure regarding frequency modulation with the highest amplitude in the lowest frequency band although note 3 has a slightly higher fundamental frequency and a longer duration (Fig. 1). At the same time both notes show an almost equal degree of a relatively large OEC expansion and only a slight difference in beak gape. These examples therefore fulfill the expectation that similar note types can be characterized by similar articulator configurations.

While it is difficult to compare note types between individuals since the four males sing different note types, it is possible to detect some similarities between birds that produce comparable note types. Bird 498 for instance produces a frequency modulated note (note 4) with an upwards sweep in the second half of the note which is comparable to note 5 in bird 705. In both cases these notes show a relatively high OEC expansion and a similar beak gape. Another comparison can be drawn in individuals 499 and 498 since they produce a rather dense harmonic stack (note 4 in bird 499, note 3 in bird 498) and in both birds these elements are produced with a relatively large beak gape, although OEC expansion varies remarkably (Fig.1). This again might indicate that the frequency pattern is more influenced by beak gape. On the other hand three of the birds produce high notes (note 1 in bird 498, note 5 in bird 499 and note 2 in bird 705) and in all three cases OEC expansion is at a minimum.

Another factor that might be influenced by beak gape and OEC expansion is amplitude and indeed three out of four birds produce the loudest note measured with the largest OEC expansion and in two cases also with a wide beak gape (Figs 1, 2; Table 4).

**Table 4:** Amplitude values of measured song elements.

	Bird 498	Bird 499	Bird 704	Bird 705
**Note 1**	62.64	61.13	58.64	54.93
**Note 2**	60.95	56.20	59.45	56.04
**Note 3**	74.09	65.76	64.70	62.69
**Note 4**	66.27	55.72	73.11	68.26
**Note 5**		61.87		69.05
**Note 6**		64.22		
**Note 7**		62.02		

Table 4 gives the amplitude values in decibel of all song elements for which beak gape and OEC expansion have been measured based on the X-ray videos.

Generally speaking the results indicate that beak gape as well as OEC expansion might act as vocal tract articulators to generate different note types within each zebra finch song. However, no clear picture concerning the specific effects of these articulators on sound modulation is emerging yet and the speaker experiment was carried out to directly address the role of beak gape and OEC expansion.

### (b) Speaker experiment

**Figure 3:**
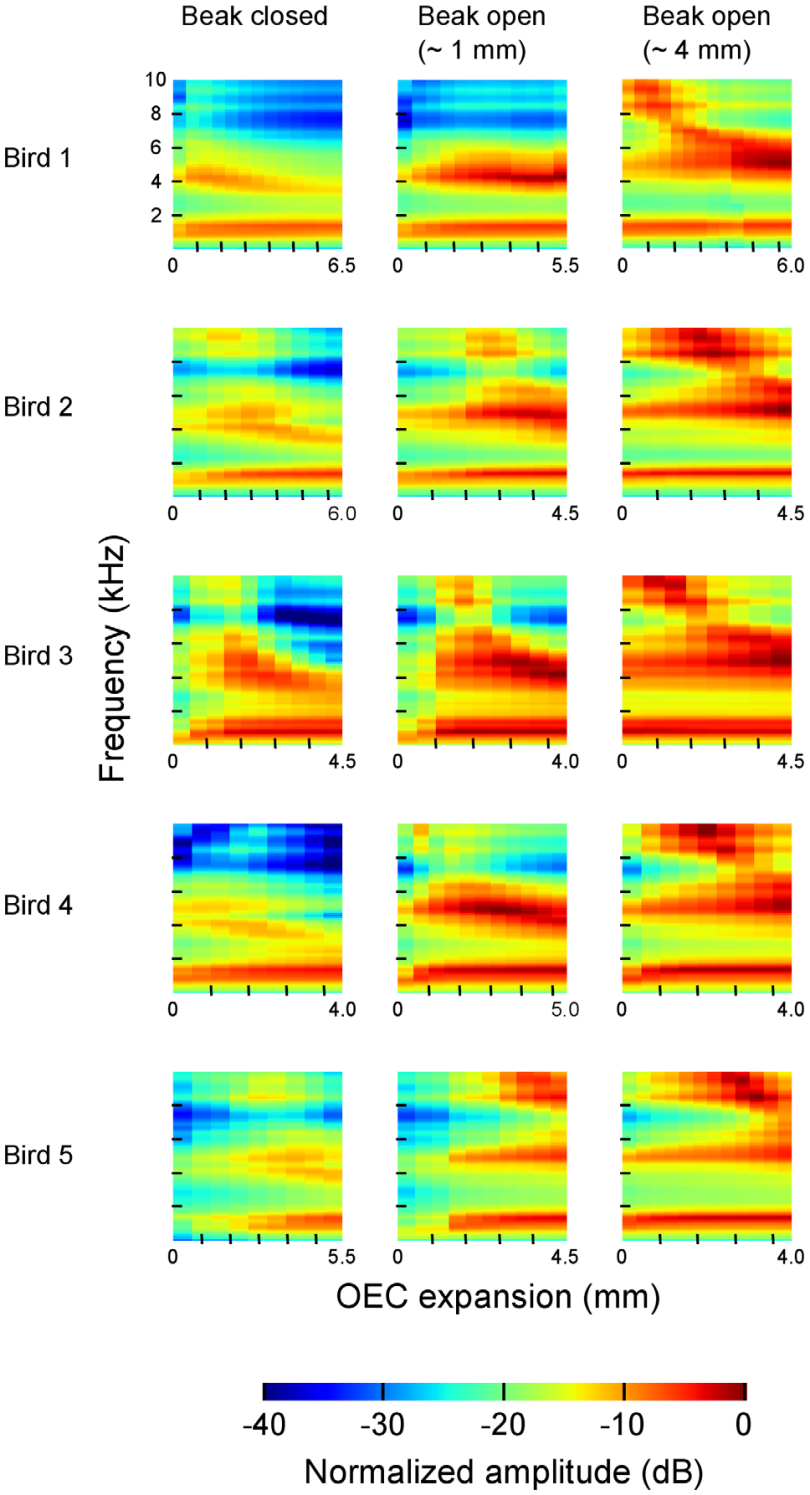
Resonance characteristics of the zebra finch vocal tract as a function of beak gape and OEC expansion. Each subplot represents the sound-energy density as a function of sound frequency and OEC expansion, which means that within a single subplot the effect of OEC expansion on the sound is illustrated. Three subplots in a row belong to one individual bird and differ from each other by the degree of beak opening. Red-orange areas represent frequencies with relatively high sound levels and therefore correspond to vocal tract resonances. This figure has been corrected for deviations in the frequency response of the speaker system. mm, millimeters; kHz, kilohertz; dB, decibel; OEC, oropharyngeal-esophageal cavity.

The results of this experiment are displayed in figure 3 which shows the effect of varying beak gape and OEC expansion on vocal tract resonances in five individual zebra finches independent of the acoustic characteristics of the syringeal sound source.

The overall frequency and amplitude modulation pattern is rather similar in all five individuals indicating a fairly specific effect of the mentioned articulators on the sound signal. The clearest and most consistent effect is given by the expansion of the OEC leading to an amplitude increase especially in the frequency region around 5 kHz which is particularly obvious at a wide beak gape, but also clearly visible when the beak is only slightly open. The total amplitude increases on average 10.9 dB when the beak is only slightly open and 8 dB when the beak is wide open. With a closed beak this effect is not so clear since amplitude first increases with OEC expansion but at a certain point drops again; in three birds even below the intensity level that was gained at position 0. Also at the other two beak gapes amplitude first increases rapidly, but drops again at a certain point although it clearly remains above the intensity level at the beginning of a series. Furthermore OEC expansion causes an energy shift towards relatively lower frequencies which again is especially obvious with a wide beak gape, but also visible in the two other conditions.

Beak gape has a strong effect on amplitude too. The wider the beak opens the louder the sound becomes with an average amplitude increase of 5.8 dB compared between a closed and a wide beak gape.

Regarding frequency range it seems that a closed beak filters frequencies above 6 kHz whereas a wide beak gape emphasizes high frequencies above 5 kHz and over a broader frequency range.

All of the nine subplots show a low prominent formant around 1.5 kHz probably mostly influenced by the resonating trachea, the dimensions of which do not change under the different articulator configurations tested in this experiment.

## Discussion

In the current study we combined observational and correlational data on song production in zebra finches with findings derived from experimental manipulations of beak gape and OEC expansion to provide insight into the mechanisms of vocal tract filtering in this species. Based on our results it seems clear that zebra finches can use both beak gape and OEC expansion as vocal articulators to filter the sound produced by the syrinx. However, while some of our results support conclusions drawn by other studies and are in line with some of the hypotheses formulated earlier, not all our findings confirm what has been discovered regarding vocal tract filtering in other bird species.

Our X-ray cinematographic imaging of singing zebra finches revealed that the expansion of the OEC is caused by a cyclical posterior-ventral movement of the hyoid skeleton which is comparable to northern cardinals [14] and white-throated sparrows [15] which both increase the volume of their oropharyngeal-esophageal cavity by cyclically moving the hyoid skeleton. In these species the OEC functions as a resonance cavity that tracks the fundamental frequency of the song which is in accordance with our data from the speaker experiment showing a downwards-shift in peak frequency with increasing OEC expansion (Fig. 3). However, it seems that in zebra finches OEC expansion also causes a general amplitude increase independent of specific frequencies which is especially obvious at a wide beak gape (Fig. 3). This does not only become apparent in the speaker experiment but also gains support by the X-ray videos since three of the birds produce the loudest note measured with the largest OEC expansion (Figs 1, 2; Table 4). In this context it is also interesting to note that reduced air sac volume in zebra finches causes sound amplitude to decrease whereas the temporal pattern of the song remains unaffected [21].

Beak gape has been shown to essentially influence frequency patterns of bird vocalizations although different studies arrive at different conclusions. The general picture emerging from the literature is that wider beak gapes correlate with higher frequencies whereas smaller beak gapes correlate with lower frequencies. This has been shown in several songbird species such as the white-throated sparrow, the swamp sparrow [8] and the song sparrow (*Melospiza melodia*) [22] but also in barnacle goose (*Branta leucopsis*) [23]. Support for a causal relationship of these observations comes from experiments in which beak gape was experimentally manipulated [12] either by immobilizing or by adding weight to the beak. In the latter case lower frequency notes were more strongly affected which in turn is consistent with findings from theoretical and experimental modeling [24] that predict a non-linear relationship between beak gape and vocal tract resonances in a way that changes at smaller beak gapes lead to relatively larger changes in vocal tract resonances.

Another study [25] confirmed in eastern towhees (*Pipilo erythrophthalmus*) the hypothesis that beak gape articulation causes significant modulation of the vocal tract filtering function. In this species frequencies between 4 and 7.5 kHz are attenuated when beak gape width is reduced. Furthermore the authors propose that towhees in particular and songbirds in general might vary beak gape as a mechanism to exclude or concentrate energy in distinct frequency bands which often results in the production of narrow-band or pure-tone sounds.

Based on the results of our speaker experiment it seems that on the one hand large beak gapes indeed sustain high frequencies (Fig. 3) and thereby partly confirm what other studies have found [8], [22] while at the same time a closed beak attenuates frequencies above 6 kHz. However, the analysis of the X-ray videos provides ambiguous results since high-frequency notes are not always produced with a large beak gape (Figs 1, 2).

Another observation made in song sparrows is that coordinated beak movements develop rather late during song learning and appear to correspond with improved tonal quality of the sound produced whereas they are not necessary for producing the acoustic fine structure of notes [22]. However, zebra finches mostly produce complex notes with energy distributed over a large range of frequencies with the fundamental frequency often being attenuated, instead of pure-tone sounds while rapid beak movements occur during the whole song. Therefore it seems unlikely that this species adjusts beak gape to improve tonal quality. In fact, Williams [10] reports a high increase in peak frequency (∼694 Hz) after beak opening movements whereas the fundamental frequency was only slightly increased (∼12 Hz). Also the average amplitude was greater after beak opening movements. Our results corroborate these findings. The speaker experiment revealed an amplitude increase with both OEC expansion as well as beak opening. At the same time peak frequency is higher when the beak is open compared to when it is closed. However we could not detect a clear effect of beak gape on peak frequency based on the X-ray data. On the one hand this might be attributed to the fact that other parameters, such as syringeal muscle activity could play a role in frequency modulation too, while on the other hand the sampling rate might be too low since only some notes could be measured per bird.

A different study found a strong positive correlation between beak gape and fundamental frequency as well as peak frequency in zebra finches in most of the individuals tested although the relationship between beak gape and fundamental frequency did not apply to harmonic stacks [11]. The authors also found a correlation between beak gape and amplitude although they conclude from their data that this relationship is likely secondary and based on a strong correlation between air sac pressure and beak gape [11]. Our X-ray data confirm that those notes produced with the largest beak gape usually have a high amplitude (Figs 1, 2; Table 4) while the speaker experiment too indicates that beak gape has a strong effect on amplitude and therefore cannot be regarded secondary.

Other structures that might be involved in vocal tract filtering include the trachea itself and glottal opening. Whereas the role of glottal opening has not been examined yet there are indications that zebra finches actively shorten the trachea at the beginning of a song bout [26]. However, modulation of tracheal length during the song motif seems to be driven by air sac pressure changes and does not clearly relate to the acoustic structure of the song. Some passerine species such as the trumpet bird (*Phonygammus keraudrenii*) exhibit elongated tracheas which are assumed to lower the pitch of the vocalizations [27] and therefore exaggerate size [28]. Figure 3 shows in every subpanel a low formant which exhibits basically no frequency modulation and is likely mostly influenced by the trachea of the birds which dimensions do not change during the experiment and therefore remains resonating at a certain frequency. However, this might be different during real vocalizations although the study mentioned above [26] did not find a clear relationship between tracheal length and song structure.

In any case it has become clear that vocal tract filtering in birds can enhance vocal complexity and serve to code biologically relevant information such as size [28], [29]. While it has been thought originally that vocal tract filtering does not apply to birdsong it is obvious nowadays that the source-filter theory of speech production can also be implemented on bird vocal communication. However, given the anatomical and physiological characteristics of the avian sound source we have to assume that the mechanisms underlying vocal production in birds are more complex than those underlying human speech production. On the one hand there is evidence that each side of the syrinx can be controlled independently in at least some songbird species [30]–[32] resulting in e.g. two-voice phenomena. On the other hand it has also been shown that the two syringeal halves may be coupled and interact with each other [33]. Moreover, a multiplicity of syringeal and respiratory muscles controlling airflow and air sac pressure play an important role in generating certain acoustic properties [34].

In summary we have shown that zebra finches can use both beak gape and OEC expansion to modulate their vocalizations to a substantial degree. However, the wide variety of different note types that these birds produce does not seem to be solely based on the interaction of these articulators but is likely to be affected also by other factors related to the sound source.

## Supporting Information

Video S1 X-ray video of a singing zebra finch
(5.69 MB WMV)
